# Parotid Gland Tumors: Molecular Diagnostic Approaches

**DOI:** 10.3390/ijms25137350

**Published:** 2024-07-04

**Authors:** Daniela Vrinceanu, Mihai Dumitru, Miruna Bratiloveanu, Andreea Marinescu, Crenguta Serboiu, Felicia Manole, Dragos Octavian Palade, Adrian Costache, Mariana Costache, Oana Patrascu

**Affiliations:** 1 ENT Department, Carol Davila University of Medicine and Pharmacy, 050474 Bucharest, Romania; vrinceanudana@yahoo.com; 2 Pathology Department, Carol Davila University of Medicine and Pharmacy, 050474 Bucharest, Romania; mirunabratiloveanu@gmail.com (M.B.); adriancostacheeco@yahoo.com (A.C.); mariana.costache@umfcd.ro (M.C.); oanamaria.patrascu@gmail.com (O.P.); 3 Radiology Department, Carol Davila University of Medicine and Pharmacy, 050474 Bucharest, Romania; andreea_marinescu2003@yahoo.com; 4 Molecular Biology and Histology Department, Carol Davila University of Medicine and Pharmacy, 050474 Bucharest, Romania; crengutas@yahoo.com; 5 ENT Department, Faculty of Medicine, University of Oradea, 410073 Oradea, Romania; manole.felicia@gmail.com; 6 ENT Department, Grigore Popa University of Medicine and Pharmacy, 700115 Iasi, Romania; drpalade@gmail.com

**Keywords:** parotid, gland, tumor, mucoepidermoid, carcinoma, molecular biology, diagnosis, genes

## Abstract

Parotid gland pathology represents a web of differential diagnoses. There are many complex cases that require extensive diagnostic tests for a complete and correct final pathology diagnosis. Currently the official classification of parotid gland tumors extends over more than 40 subtypes. We performed a query of the PubMed database regarding the use of molecular biology tests in performing a better characterization of the tumors in specific cases. By using fluorescence in situ hybridization (FISH), reverse transcription polymerase chain reaction (RT-PCR) or next-generation sequencing, the team managing complex cases can offer a personalized therapeutic solution. We review the molecular differential diagnosis according to published articles in the last 5 years for many types of parotid gland tumors ranging from benign to borderline malign tumors to malign aggressive tumors. Mucoepidermoid carcinoma is a distinct subtype of parotid malignancy that was the subject of a consistent number of articles. However, the molecular biology diagnosis techniques helped more in excluding the diagnosis of mucoepidermoid carcinoma, and probably retrospectively limiting the number of cases with this final diagnosis. In Romania, the molecular biology diagnosis is available only in limited research facilities and should receive more consistent funding that will make it available on a larger scale. The novelty of this scoping review is that we propose an algorithm for molecular differential diagnosis of the tumors that could be encountered in the parotid gland.

## 1. Introduction

The newest ESMO guidelines on salivary gland tumor management acknowledged the role of molecular diagnostics. The defining balanced translocations are evaluable on paraffin-embedded materials either by FISH, RT-PCR or next-generation sequencing (NGS). These techniques enable diagnostic differentiation between morphologically similar tumor types. Furthermore, they can identify novel driver pathways that determine tumor biology and which may be used in developing newer targeted therapy [1].

This is a scoping review that will help future diagnostic and research efforts to focus on specific molecular biology testing for differential diagnosis of parotid tumors.

Ultrasonography is a useful tool for preoperative imaging of parotid gland tumors. By using the landmark of the Stensen salivary duct, sonography can standardize the tumors in superficial or deep lobe masses [2]. Contrast enhanced ultrasonography and other techniques can visualize microvascular density. This is an important imaging characteristic for differentiating benign from malignant lesions. Neoangiogenesis is the process through which the scarce blood supply of the benign tumor is increased, and it indicates the possibility of malignant transformation [3].

CT scans with contrast media seem the most accessible imaging studies regarding parotid gland pathology. A study showed that the differential diagnosis between basal cell adenoma, myoepithelioma and a Warthin tumor can be made using the different patterns of enhancement. Actually, tumors with low cellularity and large extracellular space will retain contrast material, while tumors with high cellularity retain less contrast material with a high washout ratio [4].

MRI with diffusion weighted imaging (DWI) is a widely used technique for evaluating the rate of microscopic water diffusion in parotid gland tissue. The apparent diffusion coefficient has been proven to be a useful imaging marker for tumor diagnosis, differentiation of histologic grade, prediction of disease survival and therapeutic monitoring in various tumors [5].

This is the central tool for the management of parotid gland tumors. FNA can obtain the necessary biopsy material for performing further molecular biology testing. Exploring the mutations of *MYB*, *NTRK3*, *MAML2*, and *EWSR1* FISH might have facilitated the diagnosis of AdCC, secretory carcinoma, mucoepidermoid carcinoma, or clear cell carcinoma [6]. A French study on a cohort of 212 patients with parotid masses showed that UFNAB PPV when MRI suggested malignant tumor was 51.8% for malignant tumor, 67.7% for benign tumor and 37.5% for pleomorphic adenoma. Taking this finding into consideration, the sequence of diagnosis should focus first on MRI and secondly on FNAB [7]. The Milan System for Reporting Salivary Gland Cytopathology uses a diagnostic scheme including the following categories: (1) nondiagnostic, (2) nonneoplastic, (3) atypia of undetermined significance (AUS), (4) neoplasm (including benign neoplasms and salivary gland neoplasms of uncertain malignant potential [SUMPs]), (5) suspicious for malignancy (SM), and (6) malignant [8].

In spite of all the diagnostic procedures available for the managing surgeon, there are numerous situations of diagnostic uncertainty. As such, we aim to gather the data required for proposing a molecular differential diagnostic algorithm for parotid gland tumors with a focus on one of the most eluding tumors which is the mucoepidermoid carcinoma (MEC).

The main surgical procedure is represented by superficial parotidectomy with extension to total parotidectomy in cases with existing facial nerve paresis. There are some risks during these procedures that the patient needs to be informed about. In addition, the associated lymphadenopathy dissection and resection depends on the tumor staging [9]. One possible complication from parotid gland surgery is Frey syndrome. This can be prevented by carefully executing a modified facelift incision (MFI) approach with or without superficial musculoaponeurotic system (SMAS) reconstruction [10]. In locally advanced tumors, the resection is more complex and there is a need for complex multiple simultaneous free flaps for reconstruction. Therefore, targeted molecular treatments could be an answer to the problems of locally advanced parotid carcinoma [11].

Molecular biology tests can also be used as predictors to the possible response to chemotherapy. For example, in the case of high-grade mucoepidermoid carcinoma of the parotid gland there was a partial response to a regimen of Bevacizumab and Temsirolimus in combination with Valproic Acid [12]. One patient with oncocytic carcinoma of the parotid gland received capivasertib for an *AKT1 E17K*-mutated tumor. The treatment spanned almost 30 months confirming the safety of this compound in managing such cases [13]. A trial from 2019 on the use of Ado-trastuzumab emtansine (T-DM1) in patients with HER2-amplified tumors showed that patients with mucoepidermoid carcinoma of the parotid gland and parotid gland squamous cell cancer can have a partial response [14].

## 2. Material and Methods

**Figure 1:**
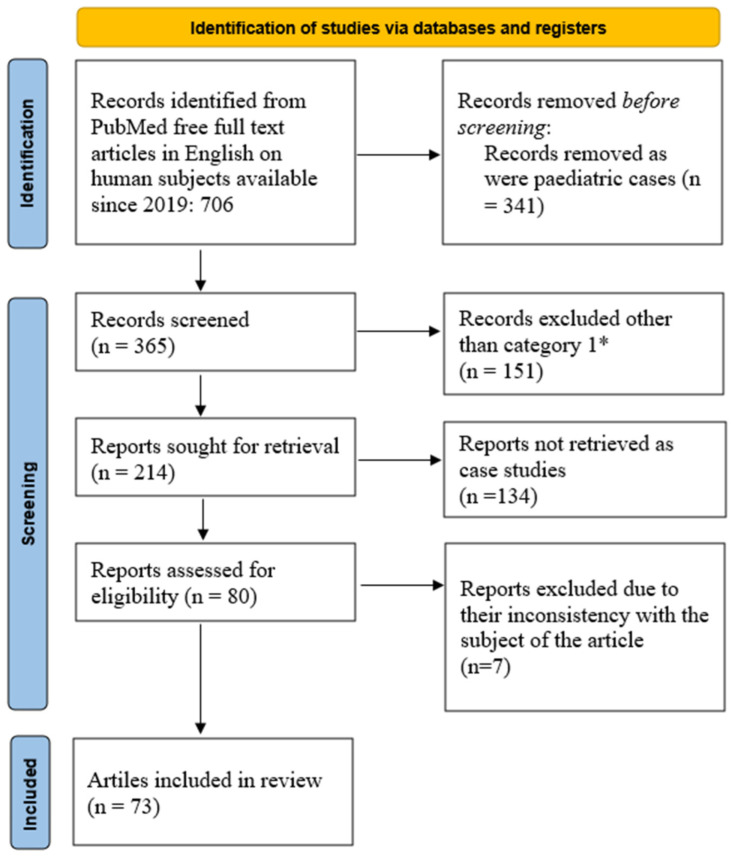
PRISMA flowchart of current scoping review. * Category 1: case reports, clinical studies, clinical trials, clinical trial protocols, clinical trial phases I to IV, comparative studies, controlled clinical trials, guidelines, meta-analyses, multicenter studies, observational studies, practice guidelines, randomized controlled trials, reviews, and systematic reviews [15].

For this scoping review, we queried Pubmed using the key words—parotid gland tumor—and restricted the results to free full text articles in English on human subjects available since 2019. The search returned a total of 706 possible articles. By further limiting the search to adult cases over the age of 19, we obtained 365 articles. Moreover, we focused on case reports, clinical studies, clinical trials, clinical trial protocols, clinical trial phases I to IV, comparative studies, controlled clinical trials, guidelines, meta-analyses, multicenter studies, observational studies, practice guidelines, randomized controlled trials, reviews, and systematic reviews, which resulted in 214 articles. In the final stage, we removed manuscripts on case studies, with the final number of articles being 80. Further manual checks excluded 7 articles due to their inconsistency with the subject of the article, Figure 1. This is one of the first endeavors in proposing an algorithm for molecular biology differential diagnosis of parotid gland tumors.

## 3. Differential Diagnosis of Parotid Gland Tumors

We will present the various specific criteria for molecular biology diagnosis in parotid gland tumors. This will help in further differential diagnosis of tumor subtypes. There are a few subtypes of parotid gland tumors that did not benefit from the use of molecular biology techniques in the differential diagnosis and could be the subject of future research.

### 3.1. Warthin Tumor

According to a retrospective study on more than 1000 cases, there is an increased incidence of Warthin tumors compared to pleomorphic adenoma. This situation is somewhat contradictory to the previous literature data [16]. Another study focused on the use of fine needle aspiration cytology (FNAC) for the diagnosis of Warthin tumors. After analyzing 1710 patients, the study concluded that a cytology positive for Warthin tumor is efficient in proposing only watchful waiting of the patient [17]. On the other hand, there is the possibility of the rapid onset of an ulceration at the level of the Warthin tumor. This can complicate with bleeding at the tumor site. These tumors are associated foci of squamous metaplasia [18]. The literature documents 15 cases of association between Warthin tumors and follicular lymphoma. The main problem resides in judging whether the lymphoma has developed inside the Warthin tumor or is a metastasis of a systemic malignancy. Occult lymphoma needs to be taken into account in such cases [19]. Recent research proposes the use of t(11;19) translocation, producing CREB-regulated transcription coactivator 1 (*CRTC1*)-*MAML2* gene fusion for differentiating a Warthin tumor from Warthin-like MEC through FISH [20].

### 3.2. Pleomorphic Adenoma

Pleomorphic adenoma is one of the most common lesions encountered in the parotid gland. It has the great potential of acquiring a malignant state due to the long term of evolution. Sometimes, there is the scenario of synchronous pleomorphic adenoma in both the parotid glands and parapharyngeal space [21]. In another situation, there can be a pleomorphic adenoma evolving synchronously with other types of pathology such as a Warthin tumor. The etiology of carcinoma ex pleomorphic adenoma is associated with the accumulation of genetic instabilities in long-standing pleomorphic adenomas because the primary tumor is present for many years, which enabled the process of malignization [22]. One variant that poses difficulties in the complete pathology characterization is the schwannoma-like pleomorphic adenoma. IHC revealed that in both the schwannoma-like and classic PA areas of the lesion, there was a strong, diffuse positivity for CK7, p63, and S-100, a patchy positivity for smooth muscle myosin heavy chains (SMMHC) and a focal positivity for calponin. In such cases, it will be necessary to analyze the molecular biology diagnosis [23]. Pleomorphic adenomas with long evolutions can undergo malignant change. Distant metastasis to the spinal cord is also possible. Although the primary tumor site was resected, the cases can present considerable morbidity due to the localization of the secondary tumors [24]. Although the etiopathogenesis of PA was initially associated with recurrent trauma at the tumor site, newer molecular biology research suggests it is associated with the proto-oncogene PLAG1, which is activated due to chromosomal aberrations that result in a fusion with genes such as CTNNB1 and LIFR [25]. These new molecular biology techniques could further help the surgeon in predicting the risks of the subsequent development of pleomorphic adenoma in complex cases and recommend more active periodic follow-up for these patients [26].

### 3.3. Solitary Fibrous Tumor

These are clearly visible, asymptomatic, non-tender, slowly enlarging swellings over the parotid area of more than 4 months duration. The immunophenotypic profile showed STAT6+, CD34+, CD99+, bcl2+, smooth muscle actin−, desmin−, citokeratin AE1/AE3−, ALK− [27]. Although these are benign rare tumors, there are around 30 cases presented in the literature with a tendency of developing malignant characteristics. In fewer cases, the MRI can show involvement of the deep lobe of the parotid gland [28]. Another study of 22 cases used FNA cytology to differentiate between various solitary fibrous tumor subtypes. Synovial sarcoma was specifically diagnosed with the support of positive TLE1 staining combined with *SS18* gene rearrangement using FISH. This molecular biology technique was a last resort in certifying the pathology of the differential diagnosis [29].

### 3.4. Desmoplastic Small Round Cell Tumor

A parotid gland is an extremely rare extra-abdominal localization of desmoplastic small round cell tumors. For a clear diagnosis, fluorescence in situ hybridization (FISH) and RT-PCR analyses were used, which revealed the presence of a gene showing fusion between exon 7 of EWSR1 and exon 8 of WT1 [30].

### 3.5. Granulomatous Pathology

Sometimes, the parotid gland can be the site of tuberculous granulomatous pathology. This type of secondary tuberculosis can pose some diagnosis challenges, due to intimate contact with the facial nerve. On other occasions, it can accompany other types of parotid gland lesions such as Warthin tumors [31]. In our query, there was no article focusing on the molecular diagnosis of this subtype of pathology. Further inquiry on this specific subject revealed a possible autoinflammatory granulomatous condition of mevalonate kinase deficiency (MKD) which is associated with painful generalized lymph node enlargement and requires testing for *MVK* 12q24 gene alteration [32].

### 3.6. Oncocytoma

This type of pathology can stem from oncocytic carcinoma. Clinically it is encountered mostly in older individuals with prior exposure to radiation. Microscopy underlines a marked mitochondrial hyperplasia [33]. An oncocytic change was also encountered in cases with a gross lipomatous tissue of the parotid gland. Mostly these are microscopic findings from resection specimens and the surgeon initially suspected a pleomorphic adenoma. Extension from the salivary ducts leads to the final diagnosis of a compound pathologic lesion such as an oncocytic sialolipoma [34]. The oncocytic sialolipoma is a mimicker of other possible pathology. In one instance, even after two core needle biopsies, the diagnosis was undetermined, and the complete surgical excision of the parotid mass was needed. Further development of molecular biology diagnoses could have helped in providing a correct diagnosis sooner [35]. Animal models propose the testing of genes responsible for the process of autophagy (allelic loss of *Beclin1* in mammary cell lines or deletion of *Atg7* in lung tumors) that result in progressing to accumulation of residues from cell metabolism. These should be explored when diagnosing future cases with oncocytomas [36].

### 3.7. Schwannoma

A study of more than 30,000 cases of tumors at the level of the head and neck revealed only 16 cases of extracranial schwannoma. One possible localization is on the trajectory of the facial nerve and inside the parotid gland, which has an increased chance of developing such tumors [37]. The function of the facial nerve is altered only in advanced stages because the tumor has a peripheral growth to the main trunk of the facial nerve. The current standard of care focuses on extracapsular dissection with preservation to the facial nerve. Given the limited number of cases, there are virtually no studies on the molecular biology diagnosis of such tumors from the parotid gland [38]. Studies from vestibular schwannoma proposed testing for CPI-17–MYPT1–merlin dysfunction. This could be a future research direction in cases with extracranial schwannoma [39].

### 3.8. Intraductal Carcinoma

Classification into the intercalated duct type and the apocrine duct type has been confirmed through molecular investigations: NCOA4-RET gene fusion is specific for the intercalated variant, and TRIM27-RET gene fusion is associated with the apocrine variant. Moreover, salivary duct carcinoma (SDC) has specific genomic alterations like HER2-neu gene amplification and hotspot mutations of PIK3CA and HRAS [40]. One of the latest studies regarding salivary ductal carcinoma analyzed the results from 70 patients. It is a genetically diverse malignancy harboring a higher number of genetic alterations including more gene rearrangements and amplifications of: *TP53*, *KIT*, *PTEN*, *SMAD4*, *ERBB2/4*, *AR*, and *APC* genes. Fine needle aspiration is able to provide the necessary tissue for performing these tests [41]. It was reported that the outcomes are worse when this type of carcinoma is present in small salivary glands than in the parotid gland. Moreover, a count of more than 11 positive metastatic lymph nodes is a reliable indicator of the severity of this tumor [42].

### 3.9. Acinic Cell Carcinoma

This is a tumor type with a low incidence, the largest study enlisted 94 cases on a span of 21 years. It has a 5-year survival rate of 95% after surgery and in a small number association with radiation therapy, but not chemotherapy. Furthermore, molecular biology tools could help in a quicker prediction of recurrence or aggressive behavior, requiring an increased aggressivity of the treatment [43]. This type of neoplasm can associate with ectopic ACTH production. In very few cases, the patients are admitted with a clinical aspect of paraneoplastic syndrome. The tumor appears as a result of deleting two tumor suppressor genes—adenomatous polyposis coli and phosphatase, and the tensin homologue [44]. Since 2010, the differential diagnosis includes a subtype of the zymogen-poor variant of acinic cell carcinoma, named as a secretory carcinoma (SC), of the parotid gland. Unfortunately, cases of SC with high-grade morphology and aggressive behavior have been identified with *ETV6-MET* fusions or simultaneous *ETV6-NTRK3* and *MYB-SMR3B* fusions [45].

### 3.10. Adenocarcinoma

Current research focuses on the better diagnosis of the cribriform adenocarcinoma of the minor salivary gland variant. From a clinical point of view, this has a higher probability of metastasis to the lymph nodes than polymorphous adenocarcinoma. Recent molecular studies indicate that rearrangements of PRKD1-3, including ARID1A-PRKD1 and DDX3X-PRKD1 gene fusions, are specific for the cribriform variant of adenocarcinoma [45].

### 3.11. Sarcomas

There is ample debate regarding the apparition de novo of carcinosarcoma or development from a previously evolving pleomorphic adenoma of the parotid gland. The new addition to the diagnostic workup is the detection of the PLAG1 mutation acquired by the pleomorphic adenoma, and thus transitions into a carcinosarcoma. However, carcinosarcoma de novo has a loss of heterozygosity of 17q21 and 9p21 [46]. In another instance, the myeloid sarcoma infiltrated a preexisting sebaceous lymphadenoma in the parotid gland. Molecular biological analysis helped in solving this diagnostic challenge and the real-time polymerase chain reaction revealed wild type genes encoding CCAAT/enhancer-binding protein *α* (*CEBPA*), nucleophosmin (*NPM1*), and the FMS-like tyrosine kinase 3-internal tandem duplication (*FLT3-ITD*). These findings also helped in hematopoietic stem-cell transplantation for efficient treatment of the patient [47].

### 3.12. Other Carcinoma

There are many subtypes of carcinomas at the level of the parotid gland. One such subtype is mammary analogue secretory carcinoma (MASC), which was encountered through a small study group. This subtype seems to have a good prognosis with only 1 from the 12 cases presenting local recurrence at 2 years follow-up [48]. Another type is nuclear protein of testis (NUT) carcinoma. These are very aggressive carcinomas with poor differentiation requiring extensive IHC differential diagnosis. Although given its name one might think that this type of tumor is encountered only in males, the latest article underlines the case of a lady under 40. FISH, reverse transcription-polymerase chain reaction (RT-PCR), cytogenetics or next-generation sequencing-based approaches determined the fusion partner *BRD3*, and more rarely *ZNF532*, *ZNF592* or *CIC* [49]. Non-sebaceous lymphadenoma can unfortunately develop a malignant change. There are fewer than 50 cases documented worldwide which showed no recurrence after surgery and no metastasis to distant organs. Flow-cytometry indicated that the tumor cells were DNA diploid, which is a sign of an initial benign nature [50]. Small cell neuroendocrine carcinoma is an extremely rare pathology with under 50 cases reported worldwide. These are very aggressive tumors that metastasize early with possible secondary lesions in the brain and in the adrenal gland. There are no studies regarding the molecular biology tests from this type of tumors and this should be a future endeavor [51]. Thymic carcinoma can arise ectopically at the level of the parotid gland. There are fewer than 10 cases documented worldwide. Although the researchers used an Illumina TruSight Oncology 500 (TSO500) hybrid-capture assay targeting 523 cancer-relevant genes for comprehensive genome profiling, there was no detection of any significant variants. The only explanation remains of an ectopic tissue undergoing malignant change [52]. Prader-Willi syndrome has a characteristic of developing multiple neoplasia. From the point of view of molecular biology diagnosis, there is a relationship between the expression of genes on the chromosome 15q11.2-q13 and malignancies. When there is a deletion, there is a higher risk of metastasizing to the parotid gland [53].

### 3.13. Metastasis to the Parotid Gland

Small cell lung cancer can metastasize to the parotid gland and there are around 10 cases documented worldwide. In this situation, there is an increased risk of facial nerve paralysis at the early onset of the metastasis. Imaging protocols reveal the primary site at the level of the lung. Obviously, metastasis presents the same molecular biology of the primary tumor [54]. In late stages of liver neoplasms, distant metastases are reported on the major salivary glands. The sites invaded by the hepatic carcinoma are most frequently the mandible and the gingiva. The major salivary glands are a sub-secondary setting [55]. Neuroendocrine tumors such as Merkel cell carcinoma (MCC) derived from the skin can lead to metastasis to the parotid gland. The correct management of such cases implies a complete examination of the skin of the patient to discover the primary site. In these cases, the oncology protocol includes adjuvant radiation therapy [56]. Papillary thyroid carcinoma metastasis to the parotid gland is also possible. This has happened even though the patient underwent total thyroidectomy and subsequent radioactive iodine therapy [57].

### 3.14. Mucoepidermoid Carcinoma

**Figure 2:**
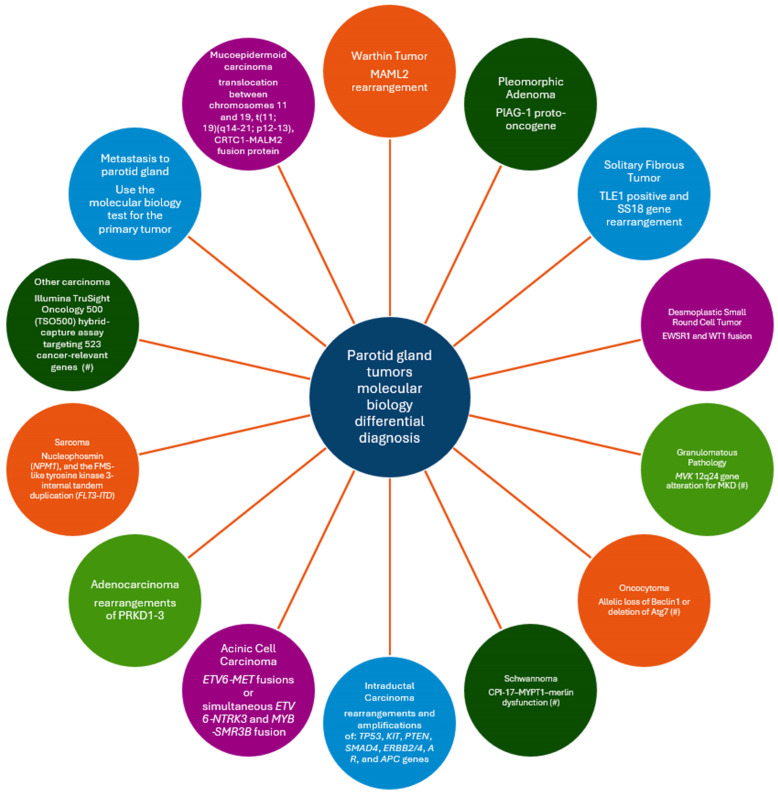
Proposal of a molecular biology differential diagnosis of parotid gland tumors. (#) indicates pathologies with only theoretical data about the possible tests to be performed.

Mucoepidermoid carcinoma (MEC) is the most common salivary malignancy, both in children and adults, representing about 10–15% of all salivary gland neoplasms [58,59]. It also represents one of the most frequently diagnosed salivary tumors in the pediatric population [60]. The most reported sites are the parotid gland and the minor salivary glands of the oral mucosa, but cases of submandibular MEC and even lung MEC have also been reported in the literature [61]. A slight female predilection is reported in most of the studies cited, although some authors postulate that male predilection is higher. Classically, the histopathological aspects include three types of cells, namely squamoid (epidermoid), intermediary and mucous secreting cells, all presenting different degrees of cytological atypia. Histologically, tumors are classified as low grade, intermediate and high-grade tumors, ranging from a cystic appearance and pseudocapsule in low grade tumors, to a more solid and infiltrative aspects in high grade cases. The overall prognostic is good, ranging from a 98% survival rate in low grade carcinomas to 67% in high grade tumors [62]. Recent analyses demonstrated a large variety of histological aspects of MEC, with columnar, oncocytic, spindle or clear cell, sclerosing variants or Warthin-like mucoepidermoid carcinoma; the latter of which was described in 2015 by Ishibashi et al. [63,64]. The diagnosis of MEC is becoming more challenging nowadays due to the histopathological variants of MEC; thus, modern tests such as FISH (fluorescent in situ hybridization) and genomic sequencing (NGS) are mandatory in some cases. In Figure 2, we present a molecular biology differential diagnosis of the tumors in the parotid gland.

## 4. Discussions

The novelty of the current scoping review is the proposed algorithm for differential diagnosis of parotid gland tumors. This seems one of the very first such endeavors on the subject. There are situations in which the pathologist faces the need for an exclusion differential diagnosis and there are some subtypes of parotid gland tumors that we found very little evidence-based data on the use of molecular diagnosis.

In this section, we will focus more on MEC due to the higher number of articles focusing on this topic. Another reason for expanding the analysis on MEC is that it seems that this subtype of parotid gland tumor requires molecular biology diagnosis in order to rule between contradicting microscopy and immunohistochemistry results.

**Figure 3:**
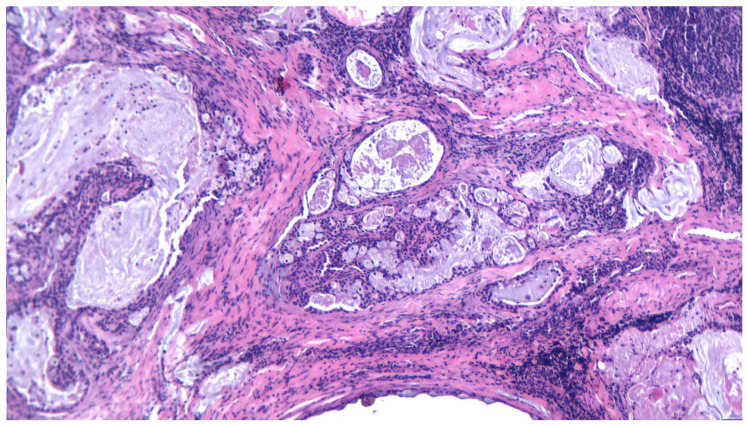
A low-grade mucoepodermoid carcinoma, with intermediate and mucous cells forming cystic structures, HE 200×.

**Figure 4:**
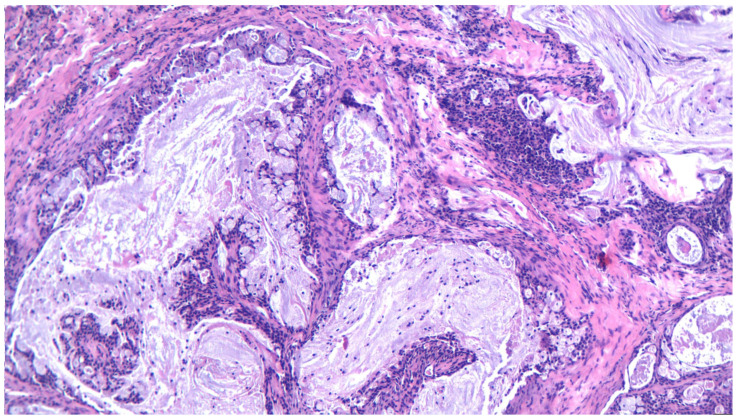
A low-grade mucoepidermoid carcinoma, with a predominance of mucous cells forming cystic structures, HE 200×.

**Figure 5:**
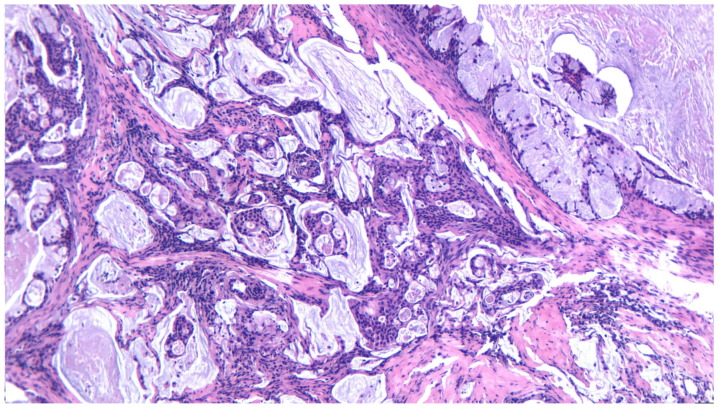
A low-grade mucoepodermoid carcinoma, with a predominance of mucous cells forming cystic structures, plus foci of mucinous-rich epithelium arranged in fused papillary structures, HE 200×.

**Figure 6:**
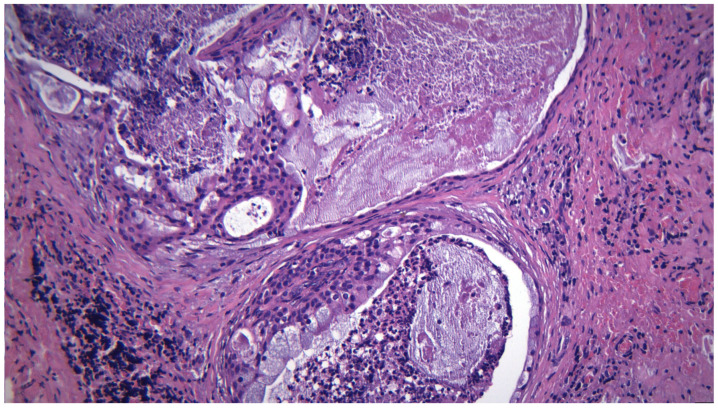
A low-grade mucoepodermoid carcinoma, with intermediate and mucous cells, detail, HE 400×.

**Figure 7:**
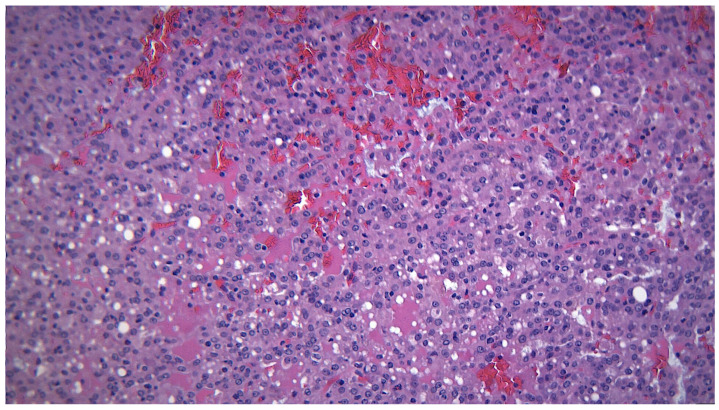
MEC with an intermediate histological grade, detail of intermediate cells, HE 400×.

MEC represents a malignant tumor arising in salivary glands, particularly in the parotid gland, but also in the submandibular gland or minor salivary glands from the hard palate. It affects a wide variety of ages, the youngest reported being a 20-month toddler [65]; however, the mean age for MEC is reported around 45 years [66]. Overall, it has a good prognosis, but high-grade tumors have the tendency to recur and metastasize to locoregional nodes, making the treatment more difficult and complex. As postulated before, diagnosing MEC is challenging due to a variety of histopathological aspects described in recent years. The classical “three cell proliferation” characteristic of MEC is sometimes shadowed by the newly discovered forms such as Warthin-like MEC, the clear cell variant of MEC, the sclerosing variant, and spindle or oncocytic cell predominance, Figure 3, Figure 4, Figure 5, Figure 6 and Figure 7.

In the literature, a technical difficulty related to the diagnosis based on FNAB is often mentioned. Theoretically, the simultaneous presence of epidermoid cells, intermediate cells and mucous cells in the presence of the mucinous background constitutes a definitive diagnosis of mucoepidermoid carcinoma, but cytology in the case of low-grade mucoepidermoid carcinoma due to the different degree of cystic fluid can lead to confusion with other lesions like mucus cysts [67].

From the point of view of an immunohistochemical study, MEC presents with peripheral MMP-9 reactivity with a high value of VEGF and CXCR4 reactivity plus galectin-3 reactivity in stromal tumor cells [68].

Regarding the prognostic, MEC can be classified into low-, intermediate and high grades based on different systems of reporting (AFIP, Brandwein et al., modified Healy), but none is unanimously accepted as they tend to underestimate or overestimate the histological grading [69]. In an attempt to better understand and classify the tumors, many studies were conducted in order to completely define the prognostic factors. In addition to the histological aspects, such as perineural invasion, surgical margins, nodal invasion, metastasis [70], tumor dimensions and deep localization, genomic alterations were regarded as prognostic factors [71,72].

In 2005, Tonon et al. discovered the gene associated with the translocation between chromosomes 11 and 19, t(11;19)(q14–21;p12–13), the CRTC1-MALM2 fusion protein, which has been confirmed in a large scale [72]. It has now been demonstrated that this genomic alternation defines more than 80% of MECs [73], while 5% of MECs are characterized by CRTC3-MALM2. The fusion protein activates the Notch pathway signaling [74,75], CREB signaling, and EGFR signaling by AREG (an EGFR ligand) especially in high grade tumors, which is useful for diagnosis and to predict the outcome of the disease. Many studies postulated that CRTC1-MALM2 protein fusion can be considered a prognostic factor as this alteration is more frequently observed in low and intermediate grade tumors, rather than high grade tumors. From 2016 [76], the gene fusion was not considered a prognostic factor; nowadays, it is considered a pathognomonic and diagnostic marker for MEC. Nevertheless, the problem of prognosis remains; therefore, novel studies targeting proteins like p53, AR or Her2neu are being conducted [74]. It is considered, for example, that AR expression in MEC, although rare, has the same importance as in salivary duct carcinoma, and that MEC with AR overexpression should be treated with androgen deprivation therapy [74]. Moreover, in addition to the fact that EGFR overexpression can be seen in 70% of high grade MECs and is associated with a poor prognosis [72], it was demonstrated that CRTC1-MAML2 positive cells are sensitive to the epidermal growth factor receptor (EGFR) tyrosine kinase inhibition pre-clinically [73] and that anti-EGFR antibodies can block MEC growth [64]. A new mutational gene, p53, is being studied in salivary tumors, namely in intermediate and high grade MECs [72]. In addition, Her2neu overexpression has been noted in high grade MECs [77], with some authors reporting as much as 80% of the analyzed tumor sensitive to HER2neu [66]. As more complex studies were performed, it is now thought that MEC can harbor as many as 180 genomic alterations in 80 unique genes [58]. Still, CRTC1/3-MALM2 protein fusion confirmed by FISH (fluorescence in situ hybridization) remains of great importance both for diagnosis and prediction outcomes of the disease.

FISH represents the unanimous accepted technique to highlight genetic alterations, but NGS (next-generation sequencing) can also be helpful. The use of these analyses should be considered in cases of MEC that are intermediate or high grade and in cases with rare histological aspects, such as Warthin-like MEC. As mentioned before, some tumors have intriguing aspects and can be indistinguishable from other neoplasia such as clear cell tumors, sarcomatoid, oncocytic or Warthin tumors. After search of the literature, many cases of MEC with special histological aspects were found. As an example, after the first description of Warthin-like MEC, rare cases have been reported [78] and all were diagnosed after FISH analyses. Moreover, cases with clear cell MEC, oncocytic differentiation or even squamoid devoid MECs are being frequently reported [79] after genomic analysis.

We have to point out that MEC, although aggressive and common in salivary gland pathology, is nonetheless considered a rare tumor, and special histological aspects are even rarer. Novel techniques such as FISH or NGS are helpful for diagnostic and prediction of the outcome of MECs, but are not ubiquitously used in everyday practice, as they are in other malignancies such as breast carcinomas. Highlighting all the genomic alterations should reflect in novel therapeutic lines. While studies are still ongoing, the preferred treatment remains surgery and adjuvant radiotherapy, depending on the status and grading of the tumors. Chemotherapy agents are being used in some cases, with polychemotherapy recommended in high grade neoplasia, but overall, with poor therapeutic outcomes [62,80].

Nonetheless, molecular biology techniques will give the great advantage of a personalized medicine approach. Molecular profiling and advancement in targeted therapies will increase the survival of parotid gland tumor patients, thus balancing the higher costs with equipment and training of staff [81].

## 5. Current Status in Romania

After a wide review of the literature, no cases of MEC examined either by FISH or NGS were reported in Romania. Although the FISH technique is available on a larger scale nowadays, special analysis for MEC tumors were not conducted as the incidence of the tumor is not very high and the cost-efficiency benefit does not sustain such analyses. In comparison, FISH is widely used for Her2neu equivoque breast carcinomas and finance-supporting projects are being carried out.

Overall, in Bucharest, there are three centers for ENT pathology, one being the University Emergency Hospital, in which only 10 cases of MECs were treated over a period of 5 years. Most of them were histopathologically and immunohistochemically confirmed, without further genomic examination. However, in the few cases with contradictory results between microscopy and immunostaining, molecular biology techniques could have been useful.

## 6. Limitations

This is a scoping review regarding the molecular diagnosis of parotid gland tumors and one limitation could be a lack of access to some articles that are not available freely online. Moreover, there were some articles that were not in English, and their accessibility is limited, although from the abstract, they seem of high quality. Another limitation is the reduced incidence and prevalence of some of the types of salivary gland tumors which reduces the chance of performing the necessary molecular diagnosis tests. In Figure 2, we underlined the fact that there are some entities in the pathology differential diagnosis for which we could not identify manuscripts about the use of molecular biology diagnosis and we proposed possible tests to be analyzed in future studies.

## 7. Conclusions

As an ancillary test, immunohistochemistry is widely used in diagnosis of salivary gland tumors, being more accessible and available. The present review enabled the proposal of a molecular biology differential diagnosis of the parotid gland tumors. As the guiding rules for the diagnosis of MECs is utterly based on the usual examination of hematoxilin-eosin slides and immunohistochemical staining, additional tests such as FISH or NGS are recommended only in special cases, such as Warthin-like MEC. In addition, the latter tests are helpful only for the diagnosis and are not currently unanimously accepted for prognostic reports. This is highlighted in the need for extensive studies that can develop accessible markers for diagnosis and prognosis (such as p53, EGFR or Her2neu), and/or to extend the availability of special tests worldwide in order to better manage the patient. As new targeted therapies emerge, molecular testing and prognostic evaluation will become mandatory for the pathological reporting of salivary gland cancers.

## Data Availability

All data are available upon request to the corresponding author.
